# 2-Phenyl­ethyl 1-thio-β-d-galactopyran­oside hemihydrate

**DOI:** 10.1107/S1600536811031667

**Published:** 2011-08-11

**Authors:** Iván Brito, László Szilágyi, Ambati Ashok Kumar, Joselyn Albanez, Michael Bolte

**Affiliations:** a Departamento de Química, Facultad de Ciencias Básicas, Universidad de Antofagasta, Casilla 170, Antofagasta, Chile; b Department of Organic Chemistry, University of Debrecen, H-4010 Debrecen Pf 20., Hungary; c Institut für Anorganische Chemie der Goethe–Universität Frankfurt, Max-von-Laue-Strasse 7, D-60438 Frankfurt am Main, Germany

## Abstract

The title compound, C_14_H_20_O_5_S·0.5H_2_O, crystallizes with two organic mol­ecules and a solvent water mol­ecule in the asymmetric unit. In both mol­ecules, the hexa­pyranosyl rings adopt a slightly distorted chair conformation (^5^ *C*_2_) with four substituents in equatorial positions and one substituent in an axial position. The main difference between the organic mol­ecules is the dihedral angle between the phenyl ring and the best plane defined by the O—C_1_—C_2_—C_3_ atoms (r.m.s deviations = 0.003 and 0.043 Å) of the hexa­pyranosyl rings [47.4 (4) and 86.5 (4)°]. In the asymmetric unit, mol­ecules are linked by two strong O—H⋯O hydrogen bonds. In the crystal, the components are linked by a total of 10 distinct O—H⋯O hydrogen bonds, resulting in the formation of a two-dimensional network parallel to the *ab* plane.

## Related literature

For synthetic methods see: Helferich & Türk (1956 ▶). For pharmacological properties of the title compound, see: De Bruyne *et al.* (1977 ▶); Choi *et al.* (2003 ▶). Gutiérrez *et al.* (2011 ▶). For puckering parameters see: Cremer & Pople (1975 ▶).
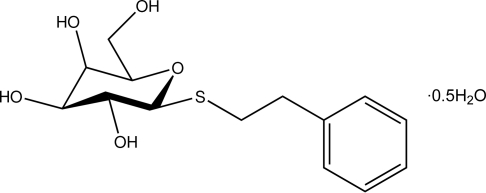


## Experimental

### 

#### Crystal data

C_14_H_20_O_5_S·0.5H_2_O
*M* *_r_* = 309.37
Orthorhombic, 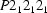

*a* = 4.8358 (4) Å
*b* = 14.8218 (16) Å
*c* = 41.390 (3) Å
*V* = 2966.6 (5) Å^3^
*Z* = 8
Mo *K*α radiation
μ = 0.24 mm^−1^
*T* = 173 K
0.20 × 0.09 × 0.08 mm

#### Data collection

Stoe IPDS II two-circle diffractometer
Absorption correction: multi-scan (*MULABS*; Spek, 200; ▶ Blessing, 1995 ▶) *T*_min_ = 0.954, *T*_max_ = 0.981
14206 measured reflections
5217 independent reflections
2395 reflections with *I* > 2σ(*I*)
*R*_int_ = 0.169

#### Refinement

*R*[*F* ^2^ > 2σ(*F* ^2^)] = 0.058
*wR*(*F* ^2^) = 0.117
*S* = 0.68
5217 reflections
377 parameters
H-atom parameters constrained
Δρ_max_ = 0.28 e Å^−3^
Δρ_min_ = −0.30 e Å^−3^
Absolute structure: Flack (1983 ▶), 2269 Friedel pairs
Flack parameter: −0.25 (16)

### 

Data collection: *X-AREA* (Stoe & Cie, 2001 ▶); cell refinement: *X-AREA*; data reduction: *X-AREA*; program(s) used to solve structure: *SHELXS97* (Sheldrick, 2008 ▶); program(s) used to refine structure: *SHELXL97* (Sheldrick, 2008 ▶); molecular graphics: *XP* in *SHELXTL-Plus* (Sheldrick, 2008 ▶); software used to prepare material for publication: *SHELXL97* and *PLATON* (Spek, 2009 ▶)’.

## Supplementary Material

Crystal structure: contains datablock(s) I, global. DOI: 10.1107/S1600536811031667/om2454sup1.cif

Structure factors: contains datablock(s) I. DOI: 10.1107/S1600536811031667/om2454Isup2.hkl

Additional supplementary materials:  crystallographic information; 3D view; checkCIF report

## Figures and Tables

**Table 1:** Hydrogen-bond geometry (Å, °)

*D*—H⋯*A*	*D*—H	H⋯*A*	*D*⋯*A*	*D*—H⋯*A*
O31—H31⋯O1*W*	0.84	1.92	2.759 (7)	179
O41—H41⋯O41*A*	0.84	1.95	2.779 (7)	169
O51—H51⋯O1*W*^i^	0.84	1.98	2.773 (6)	156
O61—H61⋯O31*A*^ii^	0.84	1.86	2.697 (7)	172
O31*A*—H31*A*⋯O61^iii^	0.84	1.92	2.650 (7)	145
O41*A*—H41*A*⋯O41^iv^	0.84	2.05	2.788 (7)	147
O51*A*—H51*A*⋯O61*A*^v^	0.84	2.10	2.785 (7)	138
O61*A*—H61*A*⋯O31^ii^	0.84	2.02	2.744 (6)	145
O1*W*—H1*WA*⋯O31^v^	0.84	2.23	2.989 (8)	150
O1*W*—H1*WB*⋯O41*A*^v^	0.84	2.43	3.270 (6)	180

Symmetry codes: (i) 
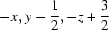
; (ii) 
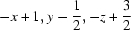
; (iii) 
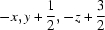
; (iv) 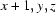; (v) 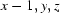.

**Fig. 1.**
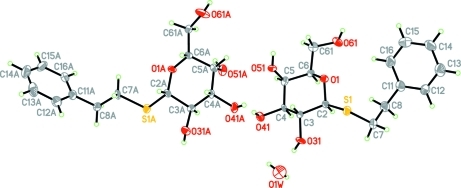
Perspective view of the asymmetric unit of the title compound, with the atom numbering. Displacement ellipsoids are shown at the 50% probability level.

**Fig. 2.**
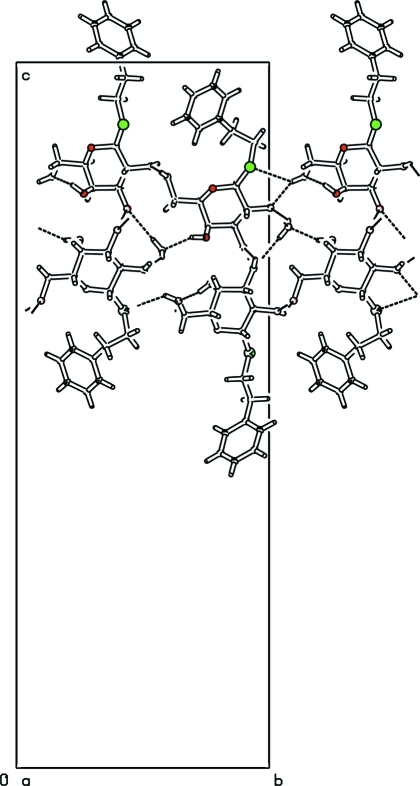
A Packing diagram, viewed down the c axis.

## supplementary crystallographic information

### Comment

2-Phenylethyl-1-thio-β-*D*-galactopyranoside is one of the most potent inhibitors of β-galactosidase (EC 3.2.1.23) (De Bruyne,*et al.*, 1977) and a radiologically labeled derivative has also been used for imaging of *LacZ* gene expression. (Choi *et al.*, 2003). It was recently found to be moderately active in tests against *Trypanosoma cruzi*, the causal agent of Chagas disease (Gutiérrez *et al.*, 2011). In the title compound, it crystallizes with two organic molecules and a solvent water molecule in the asymmetric unit, Fig. 1. In both molecules the hexapyranosyl rings adopts a slightly distorted chair conformation (^5^C_2_) (Q_T_= 0.574 (6) Å, θ= 3.4 (6)°, φ_2_ = 8(9)°; Q_T_= 0.587 (6) Å, θ= 9.2 (7)°, φ_2_ = 299 (4)° for both molecules respectively), (Cremer & Pople, 1975) with four substituents in equatorial positions and one substituent in an axial position. The main difference between the organic molecules is the dihedral angle between the phenyl ring and the best plane defined by the atoms O1/C2/C3/C4 and O1A/C2A/C3A/C4A (r.m.s deviation 0.003 Å; 0.043Å respectively), of the hexapyranosyl rings [47.4 (4) and 86.5 (4)°]. The max. deviation for the best planes of the hexapyranosyl rings are: 0.290 (7)Å and 0.050 (6) Å) for molecules A and B respectively. The mean bond distances are: C—O 1.425 (7) Å, Csp3—Csp3 1.524 (9)Å and aromatic C—C 1.386 (11) Å. In the asymmetric unit the three molecules are linked by two strong O— H···O hydrogen bonds and the crystal packing is stabilized by eight O— H···O hydrogen bonding leading to the formation of a two-dimensional network parallel to the *ab* plane, Fig. 2, Table 1.

### Experimental

1-thio-2,3,4,6-tetra-*O*-acetyl-β-*D*-galactopyranose(0.364 g,1 mmol) was dissolved in acetonitrile (2 ml) and 1-bromo-2-phenylethane (0.185 g, 1 mmol) and triethyl amine (242µl, 2 mmol) added. The reaction mixture was stirred at RT until disappearence of the starting materials (TLC, 60 min). The solvent was removed under reduced pressure, and the residue purified by column chromatography (EtOAc: hexane - 8: 2) to give 2-phenylethyl 2,3,4,6-tetra-*O*-acetyl-1-thio-β-*D*-galactopyranoside (1). Syrup, 402 mg (86%). [α]_D_ -22.9(CHCl_3_,c 0.15), Lit. (Helferich & Türk, 1956). [α]_D_ -19.2 (CHCl_3_). HR—MS:*m/z* calcd. for C_22_H_28_O_9_S[*M*+Na]^+^: 491.135. Found: 491.138. ^1^H-NMR (CDCl_3_, 500 MHz): δ 7.20–7.35 (m,5*H*, Phenyl-H); 5.44 (br.s, 1H, H-4); 5.26 (t, 1H, H-2, *J*_2,3_ 9.9 Hz); 5.03 (br.d, 1H, H-3); 4.44 (d, 1H, H-1, *J*_1,2_ 10.1 Hz); 4.10–4.20 (m,2*H*, H-6a,b); 3.90 (m, 1H, H-5); 2.92 (m, 1H, *S*-CH_2a_); 2.94 (m, 2H, Ph-*CH_2_*); 3.00 (m, 1H, *S*-CH_2 b_);2.16, 2.06, 2.04,1.99 (s, 4x3H, 4x COC*H_3_*);^13^C-NMR (CDCl_3_, 125 MHz): δ 130.5 (Phenyl-C); 85.6 (C-1); 76.5(C-5); 73.3 (C-3); 69.0 (C-4); 68.7 (C-2); 62.2 (C-6); 38.2 (Ph-*CH_2_*); 33.1 (*S*-CH_2_); 22.3 (4xCO*C*H_3_). The product (0.300 g, 0.64 mmol) was deacetylated by treatment with catalytic amount of NaOMe in methanol. The reaction mixture was stirred at room temperature until completion (TLC 20 min). After neutralization with a cation exchanger (Amberlyst 15) the solvent was removed under reduced pressure and the title molecule, 2-phenylethyl- 1-thio-β-*D*-galactopyranoside, was isolated as a white solid (MeOH: EtOAc - 2:8), 185 mg (96.3%). [α]_D_ -22.4 (MeOH, c 0.11). Lit. (Helferich &Türk,1956) [α]_D_ -32.2 (MeOH). HR—MS: *m/z* calcd. for C_14_H_20_O_5_S [*M*+Na]^+^:323.094. Found: 323.094.

^1^H-NMR(CD_3_OD, 500 MHz): δ 7.15–7.30 (m, 5H, Phenyl-H); 4.34 (d, 1H, H-1, *J*_1,2_ 9.6 Hz); 3.90 (dd, 1H,H-4, *J*_4,5_ ~ 1 Hz); 3.77(dd, 1H, H-6a, *J*_6a,6 b_ 11.5 Hz, *J*_5,6a_ 6.6 Hz); 3.71(dd, 1H, H-6 b, *J*_5,6 b_ 5.3 Hz); 3.57 (t, 1H, H-2, *J*_2,3_ 9.6 Hz); 3.53(m, 1H, H-5); 3.46 (1*H*, dd, H-3, *J*_3,4_ 3.4 Hz);2.92 (m, 1H, *S*-CH_2a_);2.95 (m, 2H, Ph-*CH_2_*); 3.02(m, 1H, *S*-CH_2 b_); ^13^C-NMR (CDCl_3_, 125 MHz): δ 131.3, 131.0, 128.9 (Phenyl-C); 88.4 (C-1); 81.5 (C-5); 77.1 (C-3);72.3 (C-2); 71.3 (C-4); 63.5 (C-6); 38.6 (Ph-*CH_2_*); 33.2 (*S*-CH_2_). Colourless single crystals suitable for X-ray analysis were obtained by slow evaporation of an aqueous solution.

### Refinement

All H atoms could be located by difference Fourier synthesis but were ultimately placed in calculated positions using a riding model with C—H = 0.95 - 1.00 Å and O—H = 0.84 Å with fixed individual displacement parameters [*U*_iso_(H) = 1.2 *U*_eq_(C) or *U*_iso_(H) = 1.5 *U*_eq_(O)].

### Crystal data

**Table:**

C_14_H_20_O_5_S·0.5H_2_O	*F*(000) = 1320
*M**_r_* = 309.37	*D*_x_ = 1.385 Mg m^−^^3^
Orthorhombic, *P*2_1_2_1_2_1_	Mo *K*α radiation, λ = 0.71073 Å
Hall symbol: P 2ac 2ab	Cell parameters from 3425 reflections
*a* = 4.8358 (4) Å	θ = 2.8–25.6°
*b* = 14.8218 (16) Å	µ = 0.24 mm^−^^1^
*c* = 41.390 (3) Å	*T* = 173 K
*V* = 2966.6 (5) Å^3^	Needle, colourless
*Z* = 8	0.20 × 0.09 × 0.08 mm

### Data collection

**Table:**

Stoe IPDS II two-circle diffractometer	5217 independent reflections
Radiation source: fine-focus sealed tube	2395 reflections with *I* > 2σ(*I*)
graphite	*R*_int_ = 0.169
ω scans	θ_max_ = 25.0°, θ_min_ = 2.8°
Absorption correction: multi-scan (*MULABS*; Spek, 200; Blessing, 1995)	*h* = −4→5
*T*_min_ = 0.954, *T*_max_ = 0.981	*k* = −17→17
14206 measured reflections	*l* = −49→47

### Refinement

**Table:**

Refinement on *F*^2^	Secondary atom site location: difference Fourier map
Least-squares matrix: full	Hydrogen site location: inferred from neighbouring sites
*R*[*F*^2^ > 2σ(*F*^2^)] = 0.058	H-atom parameters constrained
*wR*(*F*^2^) = 0.117	*w* = 1/[σ^2^(*F*_o_^2^) + (0.0007*P*)^2^] where *P* = (*F*_o_^2^ + 2*F*_c_^2^)/3
*S* = 0.68	(Δ/σ)_max_ = 0.001
5217 reflections	Δρ_max_ = 0.28 e Å^−^^3^
377 parameters	Δρ_min_ = −0.30 e Å^−^^3^
0 restraints	Absolute structure: Flack (1983), 2269 Friedel pairs
Primary atom site location: structure-invariant direct methods	Flack parameter: −0.25 (16)

### Special details

**Table:**

Geometry. All e.s.d.'s (except the e.s.d. in the dihedral angle between two l.s. planes) are estimated using the full covariance matrix. The cell e.s.d.'s are taken into account individually in the estimation of e.s.d.'s in distances, angles and torsion angles; correlations between e.s.d.'s in cell parameters are only used when they are defined by crystal symmetry. An approximate (isotropic) treatment of cell e.s.d.'s is used for estimating e.s.d.'s involving l.s. planes.
Refinement. Refinement of *F*^2^ against ALL reflections. The weighted *R*-factor *wR* and goodness of fit *S* are based on *F*^2^, conventional *R*-factors *R* are based on *F*, with *F* set to zero for negative *F*^2^. The threshold expression of *F*^2^ > σ(*F*^2^) is used only for calculating *R*-factors(gt) *etc*. and is not relevant to the choice of reflections for refinement. *R*-factors based on *F*^2^ are statistically about twice as large as those based on *F*, and *R*- factors based on ALL data will be even larger.

### Fractional atomic coordinates and isotropic or equivalent isotropic displacement parameters (Å^2^)

**Table:**

	*x*	*y*	*z*	*U*_iso_*/*U*_eq_	
S1	0.3816 (4)	0.92470 (10)	0.85163 (4)	0.0217 (4)	
O1	0.2305 (9)	0.7771 (2)	0.82113 (11)	0.0186 (11)	
C2	0.1463 (14)	0.8684 (3)	0.82448 (14)	0.0171 (14)	
H2	−0.0432	0.8697	0.8341	0.020*	
C3	0.1355 (15)	0.9128 (3)	0.79134 (14)	0.0192 (15)	
H3	0.3253	0.9114	0.7818	0.023*	
C4	−0.0578 (14)	0.8611 (4)	0.76924 (14)	0.0150 (14)	
H4	−0.2469	0.8656	0.7789	0.018*	
C5	0.0165 (15)	0.7603 (4)	0.76836 (16)	0.0206 (17)	
H5	−0.1313	0.7263	0.7566	0.025*	
C6	0.0370 (14)	0.7259 (4)	0.80286 (15)	0.0176 (15)	
H6	−0.1488	0.7321	0.8132	0.021*	
C7	0.1644 (18)	0.9413 (4)	0.88681 (16)	0.0298 (18)	
H7A	0.2706	0.9759	0.9031	0.036*	
H7B	0.0036	0.9785	0.8804	0.036*	
C8	0.0570 (17)	0.8546 (4)	0.90277 (18)	0.0335 (19)	
H8A	−0.0909	0.8706	0.9183	0.040*	
H8B	−0.0258	0.8156	0.8859	0.040*	
C11	0.2787 (17)	0.8018 (4)	0.92039 (17)	0.0302 (19)	
C12	0.3759 (19)	0.8276 (4)	0.95047 (17)	0.036 (2)	
H12	0.3035	0.8805	0.9603	0.043*	
C13	0.5745 (19)	0.7786 (5)	0.96658 (19)	0.040 (2)	
H13	0.6369	0.7982	0.9872	0.049*	
C14	0.684 (2)	0.7011 (4)	0.9531 (2)	0.044 (2)	
H14	0.8212	0.6671	0.9641	0.053*	
C15	0.588 (2)	0.6744 (5)	0.9230 (2)	0.050 (3)	
H15	0.6588	0.6205	0.9137	0.060*	
C16	0.395 (2)	0.7234 (4)	0.90633 (19)	0.042 (2)	
H16	0.3392	0.7048	0.8854	0.050*	
O31	0.0486 (12)	1.0048 (3)	0.79387 (11)	0.0352 (14)	
H31	−0.0953	1.0262	0.7855	0.053*	
O41	−0.0739 (10)	0.8992 (3)	0.73781 (10)	0.0234 (11)	
H41	0.0863	0.9052	0.7303	0.035*	
O51	0.2726 (10)	0.7497 (2)	0.75186 (12)	0.0239 (12)	
H51	0.3191	0.6951	0.7521	0.036*	
C61	0.1245 (16)	0.6275 (4)	0.80496 (16)	0.0254 (16)	
H61B	0.0055	0.5902	0.7908	0.030*	
H61C	0.3182	0.6210	0.7976	0.030*	
O61	0.0999 (11)	0.5973 (3)	0.83768 (11)	0.0276 (11)	
H61	0.2498	0.5739	0.8436	0.041*	
S1A	0.1800 (4)	0.92289 (11)	0.58806 (4)	0.0207 (4)	
O1A	0.4361 (10)	0.7963 (2)	0.62109 (10)	0.0185 (11)	
C2A	0.4278 (15)	0.8930 (4)	0.61866 (14)	0.0174 (15)	
H2A	0.6142	0.9150	0.6118	0.021*	
C3A	0.3507 (14)	0.9383 (3)	0.65050 (15)	0.0162 (14)	
H3A	0.1519	0.9264	0.6556	0.019*	
C4A	0.5327 (14)	0.9033 (4)	0.67780 (14)	0.0166 (15)	
H4A	0.7246	0.9268	0.6747	0.020*	
C5A	0.5420 (14)	0.8010 (4)	0.67805 (16)	0.0170 (15)	
H5A	0.6784	0.7805	0.6947	0.020*	
C6A	0.6377 (15)	0.7696 (3)	0.64495 (15)	0.0171 (14)	
H6A	0.8174	0.7998	0.6398	0.021*	
C7A	0.3763 (16)	0.8986 (4)	0.55177 (15)	0.0246 (16)	
H7A1	0.5560	0.9305	0.5526	0.030*	
H7A2	0.4135	0.8330	0.5504	0.030*	
C8A	0.2146 (16)	0.9289 (5)	0.52203 (15)	0.0298 (18)	
H8A1	0.0333	0.8979	0.5216	0.036*	
H8A2	0.1805	0.9946	0.5233	0.036*	
C11A	0.3715 (16)	0.9077 (4)	0.49135 (15)	0.0265 (16)	
C12A	0.5655 (18)	0.9652 (5)	0.47895 (18)	0.0323 (19)	
H12A	0.6040	1.0207	0.4896	0.039*	
C13A	0.7059 (17)	0.9425 (5)	0.45085 (19)	0.042 (2)	
H13A	0.8432	0.9822	0.4426	0.050*	
C14A	0.650 (2)	0.8640 (6)	0.43483 (19)	0.046 (2)	
H14A	0.7484	0.8492	0.4157	0.055*	
C15A	0.4537 (19)	0.8077 (5)	0.44622 (19)	0.039 (2)	
H15A	0.4146	0.7536	0.4348	0.047*	
C16A	0.3062 (18)	0.8271 (4)	0.47450 (17)	0.034 (2)	
H16A	0.1666	0.7874	0.4822	0.041*	
O31A	0.3948 (11)	1.0327 (2)	0.64734 (11)	0.0213 (10)	
H31A	0.2432	1.0585	0.6437	0.032*	
O41A	0.4250 (10)	0.9385 (3)	0.70761 (10)	0.0241 (11)	
H41A	0.5551	0.9456	0.7208	0.036*	
O51A	0.2770 (10)	0.7663 (3)	0.68599 (11)	0.0261 (12)	
H51A	0.2409	0.7220	0.6740	0.039*	
C61A	0.6753 (15)	0.6677 (4)	0.64172 (16)	0.0196 (15)	
H61D	0.5043	0.6362	0.6484	0.023*	
H61E	0.7153	0.6517	0.6190	0.023*	
O61A	0.8991 (13)	0.6415 (3)	0.66183 (13)	0.0420 (15)	
H61A	0.8688	0.5898	0.6693	0.063*	
O1W	−0.4231 (11)	1.0767 (3)	0.76658 (13)	0.0445 (14)	
H1WA	−0.5761	1.0770	0.7762	0.067*	
H1WB	−0.4623	1.0412	0.7514	0.067*	

### Atomic displacement parameters (Å^2^)

**Table:**

	*U*^11^	*U*^22^	*U*^33^	*U*^12^	*U*^13^	*U*^23^
S1	0.0215 (10)	0.0253 (7)	0.0184 (9)	0.0002 (8)	−0.0020 (8)	−0.0018 (7)
O1	0.016 (3)	0.018 (2)	0.022 (3)	0.0012 (18)	−0.004 (2)	0.0002 (19)
C2	0.016 (4)	0.020 (3)	0.015 (4)	0.003 (3)	−0.001 (3)	0.002 (3)
C3	0.029 (4)	0.015 (3)	0.014 (3)	0.003 (3)	−0.005 (3)	−0.004 (2)
C4	0.012 (4)	0.024 (3)	0.009 (4)	0.004 (3)	0.003 (3)	0.006 (2)
C5	0.020 (4)	0.025 (3)	0.017 (4)	−0.003 (3)	0.003 (3)	−0.003 (3)
C6	0.019 (4)	0.017 (3)	0.017 (4)	−0.002 (3)	−0.001 (3)	−0.003 (3)
C7	0.038 (5)	0.035 (4)	0.017 (4)	0.004 (4)	0.000 (4)	0.000 (3)
C8	0.033 (5)	0.039 (4)	0.028 (5)	0.007 (4)	0.003 (4)	0.003 (3)
C11	0.041 (5)	0.024 (3)	0.025 (5)	−0.010 (3)	0.002 (4)	0.007 (3)
C12	0.044 (6)	0.038 (4)	0.024 (4)	0.011 (4)	0.011 (4)	0.002 (3)
C13	0.051 (6)	0.040 (4)	0.030 (5)	−0.010 (4)	0.004 (5)	0.008 (4)
C14	0.063 (7)	0.028 (4)	0.041 (5)	0.004 (4)	−0.013 (5)	0.007 (3)
C15	0.066 (7)	0.033 (4)	0.053 (6)	0.011 (5)	−0.001 (6)	−0.004 (4)
C16	0.062 (6)	0.034 (4)	0.029 (5)	−0.005 (4)	−0.013 (5)	−0.004 (3)
O31	0.055 (4)	0.021 (2)	0.029 (3)	0.020 (2)	−0.018 (3)	0.001 (2)
O41	0.021 (3)	0.029 (2)	0.021 (3)	0.000 (2)	−0.005 (2)	0.0035 (19)
O51	0.034 (3)	0.013 (2)	0.024 (3)	0.0057 (19)	0.002 (3)	0.0008 (19)
C61	0.019 (4)	0.024 (3)	0.033 (4)	−0.009 (3)	0.002 (4)	0.001 (3)
O61	0.023 (3)	0.028 (2)	0.032 (3)	0.001 (2)	0.002 (2)	0.0133 (19)
S1A	0.0180 (9)	0.0286 (8)	0.0154 (9)	0.0032 (7)	−0.0022 (7)	−0.0005 (7)
O1A	0.020 (3)	0.0161 (19)	0.020 (3)	0.0050 (19)	−0.006 (2)	0.0004 (17)
C2A	0.017 (4)	0.021 (3)	0.014 (4)	0.002 (3)	−0.003 (3)	0.004 (2)
C3A	0.015 (4)	0.015 (3)	0.019 (3)	−0.003 (3)	0.000 (3)	−0.002 (3)
C4A	0.020 (4)	0.021 (3)	0.008 (3)	−0.005 (3)	−0.007 (3)	−0.002 (3)
C5A	0.014 (4)	0.019 (3)	0.018 (4)	−0.002 (3)	−0.002 (3)	0.002 (3)
C6A	0.015 (4)	0.018 (3)	0.019 (4)	0.003 (3)	−0.003 (3)	0.002 (3)
C7A	0.029 (4)	0.033 (3)	0.012 (4)	0.004 (3)	0.003 (4)	0.007 (3)
C8A	0.043 (5)	0.031 (3)	0.016 (4)	0.014 (4)	−0.003 (3)	−0.003 (3)
C11A	0.026 (4)	0.035 (4)	0.018 (4)	0.007 (4)	−0.006 (3)	0.001 (3)
C12A	0.034 (5)	0.035 (4)	0.028 (4)	0.002 (4)	−0.004 (4)	0.002 (3)
C13A	0.025 (5)	0.067 (5)	0.032 (5)	−0.001 (4)	0.004 (4)	0.016 (4)
C14A	0.042 (6)	0.074 (5)	0.022 (5)	0.023 (5)	0.000 (4)	0.000 (4)
C15A	0.044 (6)	0.042 (4)	0.032 (5)	0.013 (4)	0.005 (5)	−0.011 (4)
C16A	0.051 (6)	0.031 (4)	0.022 (4)	0.007 (4)	0.002 (4)	0.005 (3)
O31A	0.022 (3)	0.0197 (19)	0.023 (3)	−0.002 (2)	−0.008 (3)	0.002 (2)
O41A	0.032 (3)	0.027 (2)	0.014 (2)	−0.005 (2)	−0.003 (2)	−0.0025 (19)
O51A	0.029 (3)	0.028 (2)	0.021 (3)	−0.012 (2)	0.003 (2)	−0.001 (2)
C61A	0.020 (4)	0.021 (3)	0.018 (4)	0.001 (3)	0.003 (3)	0.000 (3)
O61A	0.050 (4)	0.019 (2)	0.057 (4)	0.005 (3)	−0.021 (3)	0.007 (2)
O1W	0.026 (3)	0.049 (3)	0.058 (4)	−0.015 (3)	0.005 (3)	−0.002 (3)

### Geometric parameters (Å, °)

**Table:**

S1—C2	1.804 (7)	S1A—C7A	1.813 (7)
S1—C7	1.812 (7)	O1A—C2A	1.437 (6)
O1—C2	1.420 (7)	O1A—C6A	1.443 (8)
O1—C6	1.422 (7)	C2A—C3A	1.526 (8)
C2—C3	1.522 (8)	C2A—H2A	1.0000
C2—H2	1.0000	C3A—O31A	1.421 (6)
C3—O31	1.432 (7)	C3A—C4A	1.523 (8)
C3—C4	1.516 (9)	C3A—H3A	1.0000
C3—H3	1.0000	C4A—O41A	1.437 (7)
C4—O41	1.420 (7)	C4A—C5A	1.517 (8)
C4—C5	1.537 (8)	C4A—H4A	1.0000
C4—H4	1.0000	C5A—O51A	1.419 (8)
C5—O51	1.423 (8)	C5A—C6A	1.519 (9)
C5—C6	1.519 (9)	C5A—H5A	1.0000
C5—H5	1.0000	C6A—C61A	1.527 (7)
C6—C61	1.521 (8)	C6A—H6A	1.0000
C6—H6	1.0000	C7A—C8A	1.525 (9)
C7—C8	1.535 (9)	C7A—H7A1	0.9900
C7—H7A	0.9900	C7A—H7A2	0.9900
C7—H7B	0.9900	C8A—C11A	1.512 (9)
C8—C11	1.515 (10)	C8A—H8A1	0.9900
C8—H8A	0.9900	C8A—H8A2	0.9900
C8—H8B	0.9900	C11A—C12A	1.368 (11)
C11—C12	1.385 (10)	C11A—C16A	1.419 (9)
C11—C16	1.416 (10)	C12A—C13A	1.388 (10)
C12—C13	1.376 (11)	C12A—H12A	0.9500
C12—H12	0.9500	C13A—C14A	1.366 (11)
C13—C14	1.383 (10)	C13A—H13A	0.9500
C13—H13	0.9500	C14A—C15A	1.348 (12)
C14—C15	1.386 (11)	C14A—H14A	0.9500
C14—H14	0.9500	C15A—C16A	1.400 (10)
C15—C16	1.371 (12)	C15A—H15A	0.9500
C15—H15	0.9500	C16A—H16A	0.9500
C16—H16	0.9500	O31A—H31A	0.8400
O31—H31	0.8395	O41A—H41A	0.8400
O41—H41	0.8400	O51A—H51A	0.8400
O51—H51	0.8400	C61A—O61A	1.420 (8)
C61—O61	1.431 (8)	C61A—H61D	0.9900
C61—H61B	0.9900	C61A—H61E	0.9900
C61—H61C	0.9900	O61A—H61A	0.8400
O61—H61	0.8400	O1W—H1WA	0.8394
S1A—C2A	1.799 (7)	O1W—H1WB	0.8399
			
C2—S1—C7	101.4 (4)	C2A—O1A—C6A	109.8 (4)
C2—O1—C6	111.8 (5)	O1A—C2A—C3A	112.7 (5)
O1—C2—C3	109.5 (5)	O1A—C2A—S1A	108.3 (4)
O1—C2—S1	108.7 (4)	C3A—C2A—S1A	109.7 (4)
C3—C2—S1	112.5 (4)	O1A—C2A—H2A	108.7
O1—C2—H2	108.7	C3A—C2A—H2A	108.7
C3—C2—H2	108.7	S1A—C2A—H2A	108.7
S1—C2—H2	108.7	O31A—C3A—C4A	108.5 (5)
O31—C3—C4	110.2 (5)	O31A—C3A—C2A	108.5 (5)
O31—C3—C2	110.9 (5)	C4A—C3A—C2A	110.4 (5)
C4—C3—C2	110.2 (5)	O31A—C3A—H3A	109.8
O31—C3—H3	108.5	C4A—C3A—H3A	109.8
C4—C3—H3	108.5	C2A—C3A—H3A	109.8
C2—C3—H3	108.5	O41A—C4A—C5A	111.6 (5)
O41—C4—C3	112.7 (5)	O41A—C4A—C3A	107.7 (5)
O41—C4—C5	112.2 (5)	C5A—C4A—C3A	111.3 (5)
C3—C4—C5	111.2 (5)	O41A—C4A—H4A	108.7
O41—C4—H4	106.8	C5A—C4A—H4A	108.7
C3—C4—H4	106.8	C3A—C4A—H4A	108.7
C5—C4—H4	106.8	O51A—C5A—C4A	109.7 (5)
O51—C5—C6	110.9 (6)	O51A—C5A—C6A	111.9 (5)
O51—C5—C4	108.8 (5)	C4A—C5A—C6A	108.0 (5)
C6—C5—C4	108.6 (5)	O51A—C5A—H5A	109.1
O51—C5—H5	109.5	C4A—C5A—H5A	109.1
C6—C5—H5	109.5	C6A—C5A—H5A	109.1
C4—C5—H5	109.5	O1A—C6A—C5A	109.1 (5)
O1—C6—C5	111.3 (5)	O1A—C6A—C61A	106.9 (5)
O1—C6—C61	107.4 (5)	C5A—C6A—C61A	114.7 (5)
C5—C6—C61	113.2 (5)	O1A—C6A—H6A	108.6
O1—C6—H6	108.3	C5A—C6A—H6A	108.6
C5—C6—H6	108.3	C61A—C6A—H6A	108.6
C61—C6—H6	108.3	C8A—C7A—S1A	110.0 (5)
C8—C7—S1	115.4 (5)	C8A—C7A—H7A1	109.7
C8—C7—H7A	108.4	S1A—C7A—H7A1	109.7
S1—C7—H7A	108.4	C8A—C7A—H7A2	109.7
C8—C7—H7B	108.4	S1A—C7A—H7A2	109.7
S1—C7—H7B	108.4	H7A1—C7A—H7A2	108.2
H7A—C7—H7B	107.5	C11A—C8A—C7A	111.1 (6)
C11—C8—C7	113.6 (7)	C11A—C8A—H8A1	109.4
C11—C8—H8A	108.8	C7A—C8A—H8A1	109.4
C7—C8—H8A	108.8	C11A—C8A—H8A2	109.4
C11—C8—H8B	108.8	C7A—C8A—H8A2	109.4
C7—C8—H8B	108.8	H8A1—C8A—H8A2	108.0
H8A—C8—H8B	107.7	C12A—C11A—C16A	119.6 (7)
C12—C11—C16	117.5 (7)	C12A—C11A—C8A	122.0 (6)
C12—C11—C8	122.0 (6)	C16A—C11A—C8A	118.4 (7)
C16—C11—C8	120.5 (7)	C11A—C12A—C13A	119.9 (7)
C13—C12—C11	121.8 (7)	C11A—C12A—H12A	120.1
C13—C12—H12	119.1	C13A—C12A—H12A	120.1
C11—C12—H12	119.1	C14A—C13A—C12A	121.1 (8)
C12—C13—C14	120.7 (8)	C14A—C13A—H13A	119.5
C12—C13—H13	119.7	C12A—C13A—H13A	119.5
C14—C13—H13	119.7	C15A—C14A—C13A	119.8 (8)
C13—C14—C15	118.2 (8)	C15A—C14A—H14A	120.1
C13—C14—H14	120.9	C13A—C14A—H14A	120.1
C15—C14—H14	120.9	C14A—C15A—C16A	121.6 (7)
C16—C15—C14	121.9 (8)	C14A—C15A—H15A	119.2
C16—C15—H15	119.0	C16A—C15A—H15A	119.2
C14—C15—H15	119.0	C15A—C16A—C11A	118.0 (8)
C15—C16—C11	119.9 (7)	C15A—C16A—H16A	121.0
C15—C16—H16	120.0	C11A—C16A—H16A	121.0
C11—C16—H16	120.0	C3A—O31A—H31A	109.5
C3—O31—H31	125.0	C4A—O41A—H41A	109.5
C4—O41—H41	109.5	C5A—O51A—H51A	109.5
C5—O51—H51	109.5	O61A—C61A—C6A	108.1 (5)
O61—C61—C6	109.3 (5)	O61A—C61A—H61D	110.1
O61—C61—H61B	109.8	C6A—C61A—H61D	110.1
C6—C61—H61B	109.8	O61A—C61A—H61E	110.1
O61—C61—H61C	109.8	C6A—C61A—H61E	110.1
C6—C61—H61C	109.8	H61D—C61A—H61E	108.4
H61B—C61—H61C	108.3	C61A—O61A—H61A	109.5
C61—O61—H61	109.5	H1WA—O1W—H1WB	99.1
C2A—S1A—C7A	100.7 (3)		
			
C6—O1—C2—C3	−63.2 (7)	C6A—O1A—C2A—C3A	−59.8 (7)
C6—O1—C2—S1	173.6 (4)	C6A—O1A—C2A—S1A	178.7 (4)
C7—S1—C2—O1	−109.5 (4)	C7A—S1A—C2A—O1A	−76.8 (5)
C7—S1—C2—C3	129.0 (5)	C7A—S1A—C2A—C3A	159.8 (4)
O1—C2—C3—O31	179.5 (5)	O1A—C2A—C3A—O31A	169.9 (5)
S1—C2—C3—O31	−59.5 (6)	S1A—C2A—C3A—O31A	−69.4 (6)
O1—C2—C3—C4	57.2 (7)	O1A—C2A—C3A—C4A	51.1 (7)
S1—C2—C3—C4	178.2 (4)	S1A—C2A—C3A—C4A	171.8 (4)
O31—C3—C4—O41	57.8 (7)	O31A—C3A—C4A—O41A	69.2 (6)
C2—C3—C4—O41	−179.5 (5)	C2A—C3A—C4A—O41A	−171.9 (5)
O31—C3—C4—C5	−175.2 (5)	O31A—C3A—C4A—C5A	−168.2 (5)
C2—C3—C4—C5	−52.5 (7)	C2A—C3A—C4A—C5A	−49.3 (7)
O41—C4—C5—O51	57.6 (7)	O41A—C4A—C5A—O51A	53.7 (7)
C3—C4—C5—O51	−69.6 (6)	C3A—C4A—C5A—O51A	−66.6 (7)
O41—C4—C5—C6	178.4 (6)	O41A—C4A—C5A—C6A	175.9 (5)
C3—C4—C5—C6	51.3 (7)	C3A—C4A—C5A—C6A	55.6 (7)
C2—O1—C6—C5	63.8 (7)	C2A—O1A—C6A—C5A	66.0 (6)
C2—O1—C6—C61	−171.8 (5)	C2A—O1A—C6A—C61A	−169.4 (5)
O51—C5—C6—O1	63.5 (6)	O51A—C5A—C6A—O1A	57.2 (6)
C4—C5—C6—O1	−56.0 (7)	C4A—C5A—C6A—O1A	−63.6 (6)
O51—C5—C6—C61	−57.5 (7)	O51A—C5A—C6A—C61A	−62.7 (7)
C4—C5—C6—C61	−177.1 (5)	C4A—C5A—C6A—C61A	176.5 (6)
C2—S1—C7—C8	61.9 (6)	C2A—S1A—C7A—C8A	−174.0 (5)
S1—C7—C8—C11	71.3 (7)	S1A—C7A—C8A—C11A	−178.9 (5)
C7—C8—C11—C12	77.3 (9)	C7A—C8A—C11A—C12A	−86.0 (8)
C7—C8—C11—C16	−102.6 (8)	C7A—C8A—C11A—C16A	96.4 (8)
C16—C11—C12—C13	−1.1 (12)	C16A—C11A—C12A—C13A	−3.0 (11)
C8—C11—C12—C13	179.0 (8)	C8A—C11A—C12A—C13A	179.5 (7)
C11—C12—C13—C14	−0.1 (13)	C11A—C12A—C13A—C14A	1.4 (12)
C12—C13—C14—C15	0.0 (13)	C12A—C13A—C14A—C15A	0.5 (12)
C13—C14—C15—C16	1.4 (14)	C13A—C14A—C15A—C16A	−0.7 (13)
C14—C15—C16—C11	−2.7 (14)	C14A—C15A—C16A—C11A	−0.9 (12)
C12—C11—C16—C15	2.5 (12)	C12A—C11A—C16A—C15A	2.7 (11)
C8—C11—C16—C15	−177.7 (8)	C8A—C11A—C16A—C15A	−179.6 (7)
O1—C6—C61—O61	65.0 (7)	O1A—C6A—C61A—O61A	172.2 (5)
C5—C6—C61—O61	−171.8 (6)	C5A—C6A—C61A—O61A	−66.6 (7)

### Hydrogen-bond geometry (Å, °)

**Table:**

*D*—H···*A*	*D*—H	H···*A*	*D*···*A*	*D*—H···*A*
O31—H31···O1W	0.84	1.92	2.759 (7)	179.
O41—H41···O41A	0.84	1.95	2.779 (7)	169.
O51—H51···O1W^i^	0.84	1.98	2.773 (6)	156.
O61—H61···O31A^ii^	0.84	1.86	2.697 (7)	172.
O31A—H31A···O61^iii^	0.84	1.92	2.650 (7)	145.
O41A—H41A···O41^iv^	0.84	2.05	2.788 (7)	147.
O51A—H51A···O61A^v^	0.84	2.10	2.785 (7)	138.
O61A—H61A···O31^ii^	0.84	2.02	2.744 (6)	145.
O1W—H1WA···O31^v^	0.84	2.23	2.989 (8)	150.
O1W—H1WB···O41A^v^	0.84	2.43	3.270 (6)	180.

Symmetry codes: (i) −*x*, *y*−1/2, −*z*+3/2; (ii) −*x*+1, *y*−1/2, −*z*+3/2; (iii) −*x*, *y*+1/2, −*z*+3/2; (iv) *x*+1, *y*, *z*; (v) *x*−1, *y*, *z*.

## References

[bb1] Blessing, R. H. (1995). *Acta Cryst.* A**51**, 33–38. 10.1107/s0108767394005726 7702794

[bb2] Choi, J. H., Choe, Y. S., Lee, K.-H., Choi, Y., Kim, S. E. & Kim, B.-T. (2003). *Carbohydr. Res* **338**, 29–34. 10.1016/s0008-6215(02)00359-2 12504378

[bb3] Cremer, D. & Pople, J. A. (1975). *J. Am. Chem. Soc.* **97**, 1354–1358.

[bb4] De Bruyne, C. K. & Yde, M. (1977). *Carbohydr. Res.* **56**, 153-164. 10.1016/s0008-6215(00)84247-0 18283

[bb5] Flack, H. D. (1983). *Acta Cryst.* A**39**, 876–881.

[bb6] Gutiérrez, B., Muñoz, C., Osorio, L., Ambati, A. K., Kövér, K. E., Sagua, H., Araya, J. E., Morales, P., Szilágyi, L. & González, J. (2011). *Acta Trop.* Submitted.

[bb7] Helferich, B. & Türk, D. (1956). *Chem. Ber.* **89**, 2215–2219.

[bb8] Sheldrick, G. M. (2008). *Acta Cryst.* A**64**, 112–122. 10.1107/S0108767307043930 18156677

[bb9] Spek, A. L. (2009). *Acta Cryst.* D**65**, 148–155. 10.1107/S090744490804362X PMC2631630 19171970

[bb10] Stoe & Cie (2001). *X-AREA* Stoe & Cie, Darmstadt, Germany.

